# Mediterranean diet adherence and dietary calcium intake in a group of pregnant women: Results of an Italian survey

**DOI:** 10.1002/fsn3.2233

**Published:** 2021-05-06

**Authors:** Sara Quattrini, Barbara Pampaloni, Luisella Cianferotti, Caterina Fossi, Silva Ottanelli, Giorgio Gronchi, Mirko Duradoni, Mariarosaria Di Tommaso, Valeria Dubini, Maria Luisa Brandi

**Affiliations:** ^1^ Department of Experimental and Clinical biomedical Sciences “Mario Serio” University of Florence Florence Italy; ^2^ Department of Neurosciences, Psychology, Drug Research, and Child Health (Section of Psychology) University of Florence Florence Italy; ^3^ Department of Information Engineering University of Florence Florence Italy; ^4^ Department of Health Science University of Florence Florence Italy; ^5^ Direttore Unità Funzionale Complessa – Attività Territoriali e Presidio P. Palagi USL Toscana Centro Florence Italy

**Keywords:** bone, calcium, gestational diabetes, Mediterranean diet, pulmonary hemorrhage, osteoporosis, pregnancy

## Abstract

Pregnancy is a delicate phase in woman's life that could become a risk factor for osteoporosis in pregnant women who do not meet recommended nutrient standards, especially for calcium and vitamin D. Mediterranean diet (MD) has been demonstrated to be beneficial for adequate nutrient intake. This article aims to evaluate the MD adherence and dietary calcium intake in a group of pregnant Italian women and to investigate how these are linked to each other and to fast glycemia at first trimester of pregnancy. Two hundred and seventy‐nine pregnant women were recruited at the gynecology units of two hospitals in Florence. Socio‐demographic, clinical information, and results of the first trimester blood sample analysis were collected. Two questionnaires, validated for evaluation of MD adherence and calcium intake, were administered to the pregnant women. Approximately 60% of the women had a high level of MD adherence, with a mean dietary calcium intake of 870.3 ± 335.3. In women with higher MD adherence level, fast glycemia resulted lower. Calcium intake was lower than Population Rate Intake for the Italian population (1,200 mg/daily) and was positively correlated to MD adherence score. The MD proved to be nutritious, as it was related to a higher calcium intake in this group of Italian women.

## Graphical abstract

MD adherence was positively correlated with higher calcium intake and lower fast glycemia at first trimester of pregnancy, in this population of 279 Italian pregnant women. The administration of validated questionnaires to evaluate MD adherence and calcium intake during pregnancy, through a short‐time interview (15‐20 minutes), could be inserted as a procedure of the Good Clinical Practice to prevent OP and GDM in pregnant women and to promote correct skeletal development in fetus.

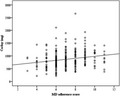

**Key points**

- MD proved to be a nutritious dietary pattern for bone health and the prevention of OP and complications (i.e., GDM) in pregnancy.
- MD adherence was positively correlated to higher calcium intake and lower fast glycemia at first trimester of pregnancy, in this population of 279 Italian pregnant women.
- The administration of validated questionnaires to evaluate MD adherence and calcium intake during pregnancy, through a short‐time interview (15–20 min), could be inserted as a procedure of the *Good Clinical Practice* to prevent OP and GDM in pregnant women and to promote correct skeletal development in fetus.

## 1 INTRODUCTION

Osteoporosis (OP) is a chronic disease that worsens the quality of life of affected patients and increases healthcare costs. It can be considered a “global problem of public health,” because the disease itself and the resulting fractures are an important cause of morbidity and mortality for millions of people worldwide (Peters & Martini, 2010).

Although OP incidence is particularly high in older women, young women may also be affected. Pregnancy itself is not a risk factor, but it could become one, if pregnant women do not intake adequate dietary nutrients and/or previous conditions have compromised the optimal achievement of peak bone mass (Società Italiana dell’Osteoporosi & del Metabolismo Minerale e delle Malattie dello Scheletro, 2016). During pregnancy and breastfeeding, calcium metabolism is subject to variations because of fetus skeleton formation. Bone resorption and calcium mobilization from bone tissue increase during the last trimester of pregnancy (Møller et al., 2012). A great deal of calcium loss also occurs through breastfeeding: in fact, calcium reserves are used for breast milk production, and the mother's skeleton could be impoverished (Møller et al., 2012), (Osteofoods, http://www.osteofoods.it/alimentazione/gravidanza‐e‐allattamento). If a woman begins and continues pregnancy and breastfeeding with a deficiency of calcium and vitamin D, she could be exposed to a high risk of OP (Ferrari et al., 2012; Hyde et al., 2017; Hyde et al., 2017; Park et al., 2017).

In order to support maternal and fetal requirements, pregnant women need a healthy diet (World Health Organization [WHO], 2016). World Health Organization (WHO) confirms that correct nutrients intake during pregnancy is achieved through the consumption of a wide variety of foods, such as vegetables, fruits, meat, fish, legumes, nuts, whole grains, and dairy products (World Health Organization, 2015).

Also, the Italian Society of Human Nutrition (Società Italiana di Nutrizione Umana, SINU) and the European Food Safety Agency (EFSA) reported that during pregnancy daily requirements of protein, polyunsaturated fatty acids (especially omega‐3 series), calcium, iron, zinc, copper, selenium, vitamin A, vitamin B group (especially B6, B12, riboflavin, thiamine), and folates increase (EFSA NDA Panel (EFSA Panel on Dietetic Products, Nutrition, and Allergies), 2012; Società di Nutrizione Umana [SINU], 2014; EFSA NDA Panel (EFSA Panel on Dietetic Products, Nutrition and Allergies), 2014, 2014; EFSA NDA Panel (EFSA Panel on Dietetic Products, Nutrition, and Allergies), 2015a; EFSA NDA Panel (EFSA Panel on Dietetic Products, Nutrition, and Allergies), 2015b; EFSA NDA Panel (EFSA Panel on Panel on Dietetic Products Nutrition and Allergies), 2015; EFSA Scientific Committee, 2015; EFSA NDA Panel (EFSA Panel on Panel on Dietetic Products Nutrition and Allergies), 2016).

The traditional Mediterranean dietary pattern is characterized by a high consumption of vegetables, fruit, legumes, nuts, and whole grains, the use of olive oil as dressing, a moderate consumption of fish, milk and dairy products, and a low consumption of meat and poultry (Rivas et al., 2013).

The Mediterranean diet (MD) was demonstrated to be associated with a reduced risk of major chronic disease (Dinu et al., 2018). As concern OP prevention, many studies reported that the incidence of fragility fractures is lower between populations with higher MD adherence (Bamia et al., 2005; Benetou et al., 2016, 2013; Byberg et al., 2016, 2015; Haring et al., 2016), although a direct cause–effect association between MD adherence and fragility fractures reduction was not demonstrated. Moreover, the consumption of Mediterranean food groups (and nutrients inside them) resulted to be fundamental for bone health (Choi & Park, 2016; De França et al., 2013; Fernández‐Real et al., 2012; García‐Martínez et al., 2014; Hayhoe et al., 2015; Mangano et al., 2015).

During pregnancy, a balanced consumption of Mediterranean food groups guarantees adequate intake of calcium, phosphorus, magnesium, proteins, and some vitamins (D and B group) and is positively associated to better bone health for newborn, especially to a higher Bone Mineral Content (BMC) during childhood and Bone Mineral Density (BMD) during adolescence (Heppe et al., 2012; Jones et al., 2000; Yin et al., 2010). Many studies highlighted that diet could be a determining factor for the development of OP in women and for the correct bone formation in the fetus (Hyde, Brennan‐Olsen, Bennett, et al., 2017; Hyde, Brennan‐Olsen, Wark, et al., 2017). In fact, a diet rich in fresh vegetables and fruit, whole bread, rice, pasta, yogurt, and breakfast cereals, and with a low intake of fried potatoes, refined sugars, white bread, processed meat, canned vegetables, and soft drinks is associated with greater bone mass in pregnant women and better bone mineralization in the fetus (Petersen et al., 2015). A cohort study by Cole et al. examined the influence of different diet styles in a population of pregnant women on bone mineralization and forearm fracture incidence in the newborn (Cole et al., 2009). Results showed that there was not a direct correlation between the mother's nutrition style and fracture incidence in the newborn, but a mother's diet poor in fruit and vegetables and rich in fats, processed meat and potatoes is associated to a higher incidence of forearm fractures during infancy (Cole et al., 2009).

Recent cohort studies demonstrated that MD could be useful also to prevent pregnancy complications, such as gestational diabetes mellitus (GDM). The diagnosis of GDM is based on different criteria: fasting plasma glucose (FPG), age, BMI (Schoenaker et al., 2016), and other risk factors (i.e., Personal history of GDM, family history of diabetes, previous macrosomia), (International Association of Diabetes & Pregnancy Study Groups Consensus Panel, 2010). Also, many studies reported that a dietary style far from the MD may contribute to the development of GDM. Assaf‐Balut et al. reported that a MD intervention in a population of pregnant women with GDM was beneficial to achieve a near‐normoglycemia and to make most pregnancy outcomes similar to those of women with normal glucose tolerance (Assaf‐Balut et al., 2017; Assaf‐Balut, Garcia de la Torre, et al., 2018; Assaf‐Balut, García de la Torre, et al., 2018). Izadi et al. explored the effect of Dietary Approaches to Stop Hypertension (DASH) and MD on the risk of GDM in a population of 460 pregnant women, of which 200 with GDM. They found that adherence to either DASH or the MD is associated with decreased risk for GDM (Izadi et al., 2016).

Many factors may contribute to higher MD adherence. Alvarez et al. performed a cross‐sectional study on a population of pregnant women in the region of Pamplona and found that women with better education, better occupation, and older age showed a greater affinity to the Mediterranean diet during pregnancy (Álvarez Álvarez et al., 2015).

The aim of this study was the evaluation of MD adherence and calcium intake in a population of 300 pregnant women, in order to investigate how these are linked to each other and to FPG at first trimester of pregnancy.

## 2 MATERIALS AND METHODS

### 2.1 Participants

Eligibility criteria were as follows: first trimester of pregnancy (10–12 gestational week) and more than 18 years of age at the time of screening, able to comprehend and speak the Italian language correctly, and resident in the metropolitan area of the study (Florence). Participation in other studies and the presence of prepregnancy pathologies (i.e., diabetes, hypertension, eating disorder, severe respiratory diseases, severe musculoskeletal disorders, inflammatory bowel diseases) were considered exclusion criteria.

The choice of this population in the first trimester of pregnancy was due to the necessity of an early involvement of pregnant women, in order to help them to follow a healthy lifestyle and to prevent OP and GDM.

In each recruitment center, a trained nutritionist illustrated the project at scheduled meetings which took place from January 2018 to January 2019 and performed the study procedures.

**FIGURE 1:**
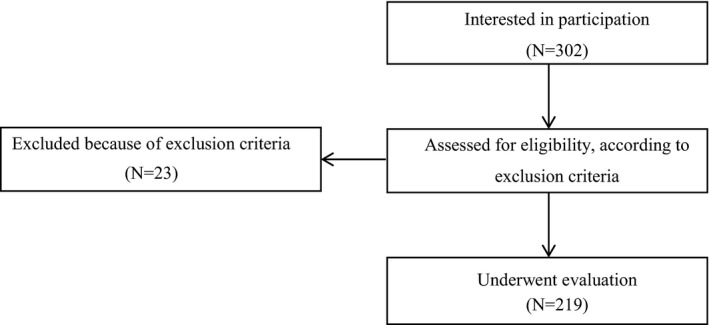
Flowchart showing a description of study design, recruitment and assessment procedures

A total of 302 subjects expressed interest in participation in the study from the two recruitment centers. Figure 1 shows the participation procedure: of the initial 302 interested subjects, 279 pregnant women were considered suitable for participation in the study.

### 2.2 General information

At the programmed informational meetings, the trained nutritionist explained the study and distributed the documents necessary for participation (patient information pack, informed consent, and consent for data handling), that were signed by the participants.

Then, the trained nutritionist performed the recruitment procedures, that included the collection of data related to: sociodemographic information (age, nationality, educational level, marital status), gestational week, weight before pregnancy, weight at the moment of visit, height, and information on lifestyle (smoking, alcoholic beverages, physical activity). Medical history was reviewed, and familiarity for pathologies (i.e., diabetes, hypertension), presence of diseases, medical treatment, supplements, and previous disorders were recorded. Finally, results of the first trimester blood analyses were recorded.

### 2.3 Dietary assessment

Among recruitment procedures, the dietary evaluation was performed through two specific questionnaires, the first for evaluation of MD adherence, and the second for evaluation of dietary calcium intake. The trained nutritionist administered both to participants.

#### 2.3.1 Questionnaire for evaluation of MD adherence

Mediterranean diet adherence was evaluated using a 13‐point score, calculated from a questionnaire, which investigated the consumption of foods and eating habits typical of the MD (Gesteiro et al., 2015). The questionnaire was previously validated by the PREDIMED Study and applied after adjustment for a population of pregnant women. For example, the question about wine consumption was omitted from the original questionnaire used in the PREDIMED Study (Martínez‐González et al., 2012), because alcoholic beverages should not be consumed during pregnancy.

Questions concerned the consumption of olive oil, vegetables, fruits, legumes, nuts, fish and white meat, and dishes with “soffritto” (defined as “sauce made with tomato and onion, leek, or garlic and simmered with olive oil”), which are considered typical MD food. Moreover, the consumption of red and processed meats, fats other than olive oil (i.e., butter, margarine), carbonated and/or sugar‐sweetened beverages, and commercial sweets or pastries (not homemade) were investigated as incorrect dietary habits, far from the Mediterranean style.

One point was assigned for each answer, according to the correct behavior, and the total score ranged from 0 to 13. The higher the score, the higher the MD adherence. Specifically, a score lower than 7 was considered “low” MD adherence, while a score equal to or higher than 7 was considered “high” MD adherence.

#### 2.3.2 FFQ for the evaluation of dietary calcium intake

A semi‐quantitative Food Frequency Questionnaire (FFQ) was administered to evaluate dietary calcium intake. It was previously validated for a population of pregnant women in a Mediterranean area (Vioque et al., 2013). The FFQ had 25 questions regarding the consumption of specific food groups, which were selected according to the MD, consumption frequency, and importance as a food source of some nutrients, especially calcium. In particular, the questions investigated the consumption of milk and dairy products, breakfast cereals, biscuits and pastries, pasta and rice, bread and derived products, potatoes, pizza, meat (red, white and processed), fish (fresh and canned), eggs, legumes, vegetables, fruits, nuts, ice cream, milk or white chocolate, and mineral water rich in calcium.

For each question, the portion size (small, medium or large—each size was specified with the quantity in grams) and the frequency of consumption (daily, weekly or monthly—it was indicated the number of times per day, per week or per month) were asked.

The assessment of nutrients intake was developed through the elaboration of a specific worksheet, using the Microsoft Excel 2010 software. The calcium content for each food was taken from the food composition Tables of the Centre of Research for Food and Nutrition (Consiglio per la Ricerca in agricoltura e l'analisi dell'Economia Agraria – CREA), (Center of Research for Food & Nutrition, http://nut.entecra.it/646/tabelle_di_composizione_degli_alimenti.html), and Food Composition Database for Epidemiological Studies in Italy (Banca Dati di composizione degli Alimenti per studi epidemiologici in Italia – BDA) of the European Institute of Oncology (Istituto Europeo Oncologico – IEO), (European Institute of Oncology, Food Composition Database for Epidemiological Studies in Italy, http://www.bda‐ieo.it/wordpress/en/).

### 2.4 Statistical analysis

Association between variables was evaluated through a Pearson correlation coefficient. The MD adherence score was correlated to BMI and dietary calcium intake. The fast glycemia was correlated to the MD adherence score, weight, and BMI.

One‐sample *t* test was used to analyze dietary calcium intake as compared to Population Reference Intake (PRI) for the Italian population, reported by the Italian Society of Nutrition (SINU) in the Levels of Reference Consumption of nutrients and energy (Livelli di Assunzione di Riferimento di Nutrienti ed energia – LARN), (Società di Nutrizione Umana (SINU), 2014). Student's *t* test was used to evaluate dietary calcium intake and fast glycemia in the two levels of MD adherence.

To compare the effect of educational status on MD adherence score, the Student's *t* test was applied.

Statistical significance was considered *p* < .05. Analyses were performed using SPSS, version 20 (SPSS).

## 3 RESULTS

### 3.1 General information

The sociodemographic characteristics and lifestyle habits of the whole population are summarized in Table 1. The mean age of participants was 34.5 ± 5.3, and the gestational age was 10.3 ± 1.1 weeks.

**TABLE 1:** Socio‐demographic characteristics and lifestyle habits of the whole population (*N* = 279)

Variables	Total sample (*N* = 279)	*SD*
Age (years – mean)	34	±5.3
Weight (kg – mean)	60.7	±10.6
BMI (mean)^a^	22.3	±3.6
Gestational age (weeks – mean)	10.3	±1.1
Fast glycaemia (mg/dl – mean)^b^	82.8	±7.1
Nationality (%)	Italian: 90.3%	
	European: 6.5%	
	Not European: 3.2%	
Marital status (%)	Unmarried: 7.2%	
	Married/cohabitant: 90.7%	
	Separated/Divorced: 2.2%	
Educational status (%)	Middle school degree: 5%	
	High school degree: 38.4%	
	Academic degree: 53.4%	
	Post‐academic degree: 3.2%	
Smoking (%)	Yes: 3.9%	
	No: 96.1%	
Alcoholic beverages (%)^c^	Yes: 6.5%	
	No: 99.6%	
Physical activity (%)	Yes: 28%	
	No: 72%	

^a^ *N* = 276.

^b^ *N* = 255.

^c^ *N* = 278.

Most of the women were of Italian nationality (90.3%), married, and/or cohabitant (90.7%) and had an academic degree (53.4%). During the first trimester of pregnancy, mean weight was 60.7 ± 10.6 kg and mean BMI was 22.3 ± 3.6, which meant normal weight. In a subgroup of 255 women, who had had blood tests, the mean fast glycaemia was 82.8 ± 7.1 mg/dl.

The 96.1% of the women answered that they did not smoke, 93.2% did not drink alcoholic beverages, and only 28% said that they practiced regular physical activity (i.e., walking, gym exercises, swimming). The mean minutes per week of physical activity was 178.1 ± 132.3, which is in line with World Health Organization (WHO) recommendations (World Health Organization, 2010).

Only 14.3% of the women said that they were taking vitamin D and 44.4% were taking pharmacological therapies, such as progesterone, levothyroxine, heparin, and acetylsalicylic acid.

### 3.2 Dietary habits

The quality of nutrition was examined in terms of answer frequencies to the FFQ questions regarding consumption of foods. Concerning the common dietary sources of calcium, 63.8% of the women consumed milk, 73.1% yogurt, and 96.1% cheese. Only 15.8% answered that they drank natural mineral water rich in calcium (calcium content in water ≥ 15, mg/L).

#### 3.2.1 MD adherence score

The questionnaire for the evaluation of MD adherence showed that the mean score was 7.1 ± 1.7 and 59.5% of women had a “high” level of MD adherence. The sociodemographic characteristics and lifestyle habits of women with low and high MD adherence are shown in Table 2.

**TABLE 2:** Socio‐demographic characteristics and lifestyle habits of women with low and high MD adherence

Variables	Women with low MD adherence (*N* = 113)	*SD*	Women with high MD adherence (*N* = 166)	*SD*
Age (years – mean)	33.8	±5.7	35.0	±4.9
Weight (kg – mean)	61.1	±11.5	60.4	±10.0
BMI (mean)	22.7^a^	±3.8	22.1^b^	±3.5
Gestational age (weeks – mean)	10.3	±1.1	10.3	±1.1
Fast glycemia (mg/dl – mean)	83.9^c^	±7.5	82.0^d^	±6.7
Nationality (%)	Italian: 90.3%		Italian: 90.4%	
	European: 4.4%		European: 7.8%	
	Not European: 5.3%		Not European: 1.8%	
Marital status (%)	Unmarried: 8.8%		Unmarried: 6%	
	Married/cohabitant: 87.6%		Married/cohabitant: 92.8%	
	Separated/divorced: 3.5%		Separated/divorced: 1.2%	
Educational status (%)	Middle school degree: 8%		Middle school degree: 3%	
	High school degree: 41.6%		High school degree: 36.1%	
	Academic degree: 47.8%		Academic degree: 57.2%	
	Post‐academic degree: 2.7%		Post‐academic degree: 3.6%	
Smoking habit	Yes: 5.3%		Yes: 3%	
	No: 94.7%		No: 97%	
Alcoholic beverages consumption	Yes: 3.5%		Yes: 8.4%	
	No: 96.5%		No: 91%^e^	
Physical activity	Yes: 23.9%		Yes: 30.7%	
	No: 76.1%		No: 69.3%	

^a^ *N* = 112.

^b^ *N* = 164.

^c^ *N* = 104.

^d^ *N* = 151.

^e^ *N* = 165.

A Student's *t* test between MD adherence score in women with a high school degree and women with an academic degree was applied. There was a significant difference between the two educational statuses: women with a higher educational status also had a higher MD adherence score (*t*(254) = −2.26, *p* = .025).

The MD adherence score also had a significant inverse correlation with the BMI, as shown from the Pearson correlation coefficient. The Pearson correlation value between MD adherence score and BMI was −0.126 (*p* = .036).

#### 3.2.2 Dietary calcium intake

The mean dietary calcium intake from the FFQ was 870.3 ± 335.3. A one‐sample *t* test was applied to compare it with the PRI for the Italian pregnant population (1,200 mg per day): dietary calcium intake resulted to be significantly lower than PRI (*t*(278) = −16.42, *p* < .001).

**FIGURE 2:**
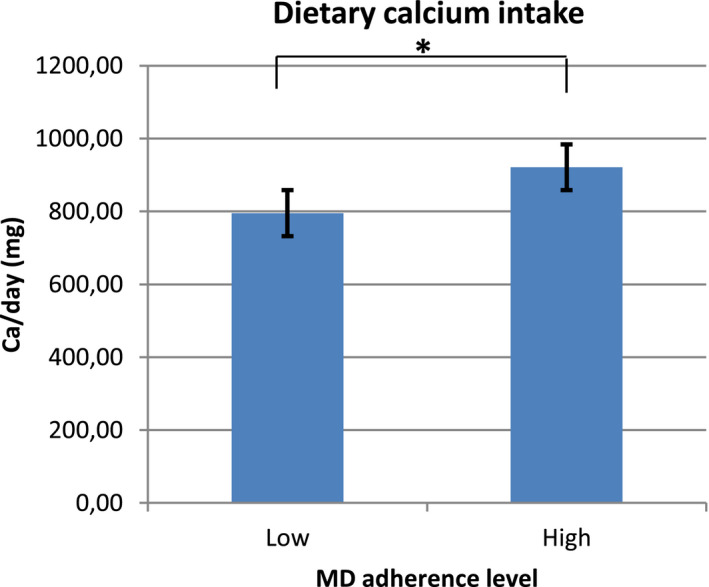
Dietary calcium intake compared in the two groups of MD adherence (Student's *t* test; *p = .002)

The dietary calcium intake was analyzed in the two groups of MD adherence level, performing a Student's *t* test. There was a significant difference between the two levels of MD adherence: women with higher MD adherence also had a higher intake of calcium (*t*(277) = −3.13, *p* = .002) (Figure 2).

**FIGURE 3:**
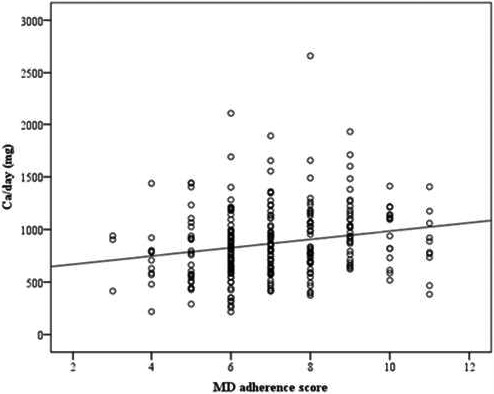
Scatter plot of Pearson's correlation between dietary calcium intake (mg/day) and MD adherence score (*p* = .001)

The MD adherence score was also significantly correlated to calcium intake (Figure 3). The Pearson correlation value between MD adherence score and dietary calcium intake was 0.199 (*p* = .001).

### 3.3 Fast glycemia and anthropometric characteristics in relation to dietary habits

**FIGURE 4:**
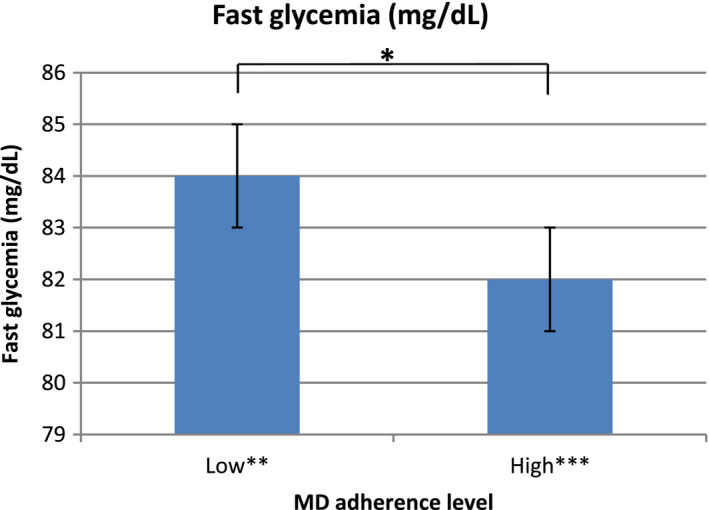
Fast glycemia compared in the two MD adherence levels (Student's *t* test; *p = .029). **N = 104; ***N = 151

Mean fast glycemia was evaluated in the two groups of MD adherence (low and high) through a Student's *t* test. A significant difference was found in the two MD adherence levels concerning fast glycemia, as shown in Figure 4 (*t*(253) = 2.19, *p* = .029).

Fast glycemia was also correlated to the MD adherence score, weight, and BMI, as results from the Pearson correlation coefficient showed significant correlations between fast glycemia and other variables, (Table 3). In particular, the higher the MD adherence score, the lower the fast glycemia. On the contrary, women with higher weight and BMI also had higher levels of fast glycemia.

**TABLE 3:** Pearson correlation between fast glycaemia and MD adherence score, weight and BMI

	Fast glycemia	*p*
MD adherence score	−0.134	.032
Weight (kg)	0.194	.002
BMI	0.229	<.001

## 4 DISCUSSION

This study investigated dietary behaviors of a group of pregnant Italian women at the beginning of pregnancy (Gestational week = 10.3 ± 1.1). In particular, the relationship between MD adherence level and calcium intake was evaluated, with respect to fast glycaemia in the first trimester.

Results showed that 59.5% of women had a high level of MD adherence, but the score was not high (7.1 ± 1.7). In fact, the high level of MD adherence is assigned for a score between 7 and 13. In this population, it is emerged that women with a higher MD adherence level were older (35.0 ± 4.9) than the ones with a low MD adherence level (33.8 ± 5.7) and had a lower weight (60.4 ± 10.0 versus 61.1 ± 11.5) and BMI (22.1 ± 3.5 versus 22.7 ± 3.8). Most were married or cohabitant (92.8%) and nonsmokers (97%). Alcoholic beverage consumption was lower in women with a low MD adherence level (3.5%), compared with high MD adherence level women (8.4%).

Educational status was higher in high MD adherence level women: 60.8% had an academic or post‐academic degree, compared with 50.5% of low MD adherence level women. Educational status also influenced the total MD score: the higher the degree, the higher the total MD score. In particular, a significant difference in the MD score emerged between women with a high school degree (6.85 ± 1.41) and women with an academic degree (7.32 ± 1.80). These results are comparable to others in the literature (Pavičić Žeželj et al., 2018). As reported by Cano‐Ibáñez N. et al. in their cross‐sectional study on subjects with metabolic syndrome, educational level was a variable that significantly influenced a better micronutrient density, as the dietary behavior is healthier in people with a higher educational background and higher socioeconomic status (Cano‐Ibáñez et al., 2019). Many factors could influence the adherence to this dietary pattern. In a cross‐sectional study on 1,051 pregnant women, there were significant differences in the MD score on the basis of educational level, occupation, and age, with women with a higher educational status exhibiting a greater affinity to the MD (Álvarez Álvarez et al., 2015).

The MD adherence score had also a significant inverse correlation with BMI. This result could be explained in different ways. In the first trimester of pregnancy, a higher BMI may be due to a lower MD adherence score, but a lower MD adherence score could also be related to a higher BMI. To our knowledge, higher BMI at the beginning of pregnancy explains the effect of the MD on the risk of developing maternal pregnancy complications, such as GDM and HDP, representing a variable that directly influences dietary choices and, consequently, a healthier status in pregnant women (Schoenaker et al., 2016).

The MD has demonstrated many positive effects on pregnancy outcomes, whether this precedes pregnancy or occurs in pregnant women, and this is particularly true for the prevention of GDM (Amati et al., 2019; Olmedo‐Requena et al., 2019; Osorio‐Yáñez et al., 2017). In our group, results showed that fast glycaemia at week 10–12 of gestation was significantly different in the two levels of MD adherence, with a higher level of MD adherence resulting in a lower fast glycemia. This parameter could be predictive for GDM and having a low value of fast glycemia at first trimester could be preventive (Assaf‐Balut, Garcia de la Torre, et al., 2018; Assaf‐Balut, García de la Torre, et al., 2018; Rani & Begum, 2016). Not surprisingly, a lower fast glycemia resulted to be positively correlated in a statistically significant way to lower weight and BMI.

Another important aspect that emerged from the results is the link between MD adherence and calcium intake. The semi‐quantitative FFQ allowed to evaluate the general quality of nutrition and dietary calcium intake. The importance of exploring dietary patterns in pregnant women is widely recognized, in order to examine food choices and evaluate macro‐ and micronutrients deficiencies (Chen et al., 2016). The mean dietary calcium intake from the FFQ was 870.3 ± 335.3: it was significantly lower than PRI (1,200 mg per day), demonstrating that more information campaigns on this topic are needed to promote maternal and fetus bone health (Hyde, Brennan‐Olsen, Bennett, et al., 2017; Hyde, Brennan‐Olsen, Wark, et al., 2017). Regarding calcium food sources, women said they consumed milk (63.8%), yogurt (73.1%), and cheese (96.1%), but only 15.8% reported drinking calcium‐rich mineral waters. At a time when calcium requirements are higher, a dietary choice in alternative and/or in addition to dairy products, such as calcium‐rich mineral waters, could be useful for meeting those requirements (Vannucci et al., 2018).

A relationship between MD adherence and calcium intake emerged in our data. In fact, there was a significant difference between the two levels of MD adherence and calcium intake: women with higher MD adherence had also a higher intake of calcium. A positive correlation between total MD adherence score and calcium intake was also statistically significant. This is a fascinating and innovative aspect in research on bone health for pregnancy. Although many variables could influence dietary choices and nutrient intake, these data may prove that a beneficial Mediterranean dietary pattern could also be helpful for the intake of micronutrients that are important during pregnancy (i.e., calcium). Attention to healthy food choices could lead to higher amounts of these essential nutrients. In fact, the reduction in bone mineral density during pregnancy is on average 2%–5%, and a period of 6 months breastfeeding results in the loss of a further 1.5%–4% (Ferrari et al., 2012). Many studies have shown that a mother's lifestyle, body composition, and nutrition are crucial for her bone health, for OP prevention and for the well‐being of the fetus and the newborn (Langdahl, 2017; Marangoni et al., 2016).

Finally, from data collected about lifestyle behavior, it's emerged that only 28% of women practiced regular physical activity (i.e., walking, gym exercises, swimming). Regular exercise during pregnancy has been shown to be beneficial not only for cardiac and pulmonary functions, but also to improve muscle, skeletal, and psychological well‐being (Oliveira et al., 2017). The role of PA in gestational weight control is widely recognized, especially for those with an exercise frequency of 3 times per week with a duration of 30 to 45 min (Wang et al., 2019). Furthermore, PA proved to be fundamental for the OP prevention (Moreira et al., 2014; Xu et al., 2016), also during pregnancy. A case–control study by Hadji et al. reported that pregnant women with transient osteoporosis of the hip (TOH) had a frequency of immobilization threefold higher compared with the control group (Hadji et al., 2017).

The limitations are due to the study design: this is an observational study and a cause‐effect association between MD, calcium intake and OP prevention could not be established. Moreover, the use of a FFQ may be subjected to recall bias. Unfortunately, in this study serum vitamin D levels and their potential correlation with glycaemia were not evaluated. Further clinical studies could be useful to deepen this argument, in order to investigate whether Vitamin D may influence metabolic variables, as fast glycemia. Nevertheless, in this study, two hundred and seventy‐nine women were recruited and the sample size was in line with previous studies with the same characteristics (Groth et al., 2017; Hyde, Brennan‐Olsen, Bennett, et al., 2017; Hyde, Brennan‐Olsen, Wark, et al., 2017; Park et al., 2017). On the basis of this literature, the size of effect was small (Cohen's *d* = 0.02), the significance level was for *p* < .05 and the test power resulted to be 0.93, with this sample size.

Moreover, the administrated questionnaires are validated and reproducible through a short‐time interview (15–20 min). This allowed to be inserted as a procedure of the *Good Clinical Practice*.

## 5 CONCLUSIONS

In conclusion, pregnancy is a delicate period in a woman's life, during which dietary requirements are modified. The evaluation of nutritional habits among pregnant women could be useful to study eventual nutrient deficiencies and to support a healthy pregnancy (Marangoni et al., 2016).

The prevention of chronic diseases, such as osteoporosis, takes place through lifestyle choices. Intervention activities that are focused on nutritional changes and exercise are in the best interest for human health (Perreault et al., 2018).

Mediterranean diet has been proven to be a wholesome dietary pattern and a useful nutrition to prevent bone loss, typical of pregnancy (Ferrari et al., 2012). In fact, the higher was MD adherence, the higher was calcium intake, in this group of Italian women.

MD adherence was also inversely correlated to fast glycaemia: women with a higher level of MD adherence had also a lower fast glycaemia. This could be fundamental for the prevention of pregnancy complications, such as GDM.

Good nutrition for pregnant women is necessary for the improvement of maternal and newborn health. Pregnancy itself it could become a timely phase for correcting dietary choices and improving one's lifestyle. Future interventional clinical trials may clarify the link between MD, calcium intake and OP prevention in pregnancy.

## CONFLICT OF INTEREST

B.P., S.Q., G.G., and F.G.: None. M.L.B. has received honoraria from Amgen, Bruno Farmaceutici, Calcilytix, Kyowa Kirin; Academic grants, and/or speaker: Abiogen, Alexion, Amgen, Bruno Farmaceutici, Echolight, Eli Lilly, Kyowa Kirin, MSD, NPS, Servier, Shire, SPA, Theramex; Consultant: Alexion, Bruno Farmaceutici, Kyowa Kirin, Servier, Shire.

## ETHICAL APPROVAL

This was a perspective, observational, multi‐centric, spontaneous, and no‐profit study, that involved pregnant subjects at clinics of the USL Toscana Centro at the Piero Palagi Hospital and the Careggi Hospital, in Florence, Italy. The study was approved by the Institutional Review Board (Comitato Etico Area Vasta Centro, Azienda Ospedaliera Universitaria Careggi, Florence, Italy) [number: 11538_OSS]. The Ethics Committee verified the conformity to the *Good Clinical Practices* and to the Declaration of Helsinki in each research centre. All patients gave informed consent for participation in the study and consent for data publication.

## Data Availability

The data presented in this study are available on request from the corresponding author.
